# Bovine Tuberculosis in a Rural Greek Area: Within-Herd Prevalence and Risk Factors in 30 Tuberculosis Skin Test-Positive Herds

**DOI:** 10.3390/ani16182947

**Published:** 2026-09-19

**Authors:** Ioannis Tsakmakidis, Theofilos Kantzoglou, Zacharenia Kyrana, Maria V. Alvanou, Georgia Koutouzidou, Katerina Melfou, Ioannis A. Giantsis

**Affiliations:** 1 Faculty of Agricultural Sciences, University of Western Macedonia, 53100 Florina, Greece; gtsakmakidis@yahoo.gr (I.T.); lakiteo27@gmail.com (T.K.); mariaalvanou7@gmail.com (M.V.A.); gkoutouzidou@uowm.gr (G.K.); kmelfou@uowm.gr (K.M.); 2 Veterinary Directorate of Western Macedonia, 50132 Kozani, Greece; 3 Department of Animal Science, Faculty of Agriculture, Forestry and Natural Environment, Aristotle University of Thessaloniki, 54621 Thessaloniki, Greece; kyranaze@agro.auth.gr

**Keywords:** *Mycobacterium bovis*, intradermal tuberculin test, cattle health, zoonotic pathogens, rural livestock systems

## Abstract

Bovine tuberculosis (bTB) is a zoonotic disease of major importance for cattle health, farm productivity, and public health. This study aimed to characterize the epidemiological features and assess their associations with farm characteristics of bovine tuberculosis-positive herds in a high-prevalence area of Greece. Apparent animal-level positivity for MBTC infection was evaluated in cattle from 30 infected farms in the Regional Unit of Florina, and patterns associated with positivity were explored. A total of 1464 animals were examined using the single intradermal tuberculin test, of which 563 were positive (38.46%). All skin test-positive animals were confirmed by PCR. After accounting for herd-level clustering, age group had the highest influence on positivity. Differences in positivity associated with production systems, husbandry categories, and co-infection with bacteria such as *Mycoplasma bovis*, which was identified in 12 animals, were observed but were not significant. Large differences were observed in positivity associated with proximity to the cross-border area, which, although not significant, indicated an interesting trend, suggesting that this parameter may be a potential risk factor for tuberculosis due to the different regulations. Overall, the findings indicate substantial variation between farms and highlight conditions linked with positivity within the infected herds. The results underscore the need for representative surveillance and improved monitoring approaches to better understand infection patterns and support evidence-based control strategies in similar livestock systems in rural areas.

## Simple Summary

In this study, 1464 animals from 30 cattle farms confirmed to be positive for bovine tuberculosis during a surveillance process were examined using the single intradermal tuberculin test. Apparent animal-level positivity for MBTC infection was 38.46%, whereas within-herd prevalences ranged from 4% to 100% across the sampled farms. All skin test reactors were confirmed by PCR. Based on multilevel logistic regression, age was the most significant factor associated with apparent positivity, and differences in apparent positivity were also observed among animal and farm characteristics, with a noteworthy observation regarding proximity to cross-border areas, which, although not significant, indicated a trend toward positivity. These findings describe the variation in apparent positivity among infected herds and underline the importance of considering herd-level clustering when interpreting results from convenience-selected farms.

## 1. Introduction

Tuberculosis (TB) is a serious disease that affects numerous mammalian species, including wildlife, livestock and humans. Among other serious and fatal diseases, tuberculosis has been identified over the last 200 years as the most severe killer of the human population, being responsible for the deaths of 1 billion people [1,2]. Currently, it is considered the second-leading cause of death due to a single infection, with approximately 10 million new infections and 1.5 million deaths each year worldwide [3]. The causative agents of tuberculosis belong to the *Mycobacterium tuberculosis* complex (MTBC), which includes *M. tuberculosis*, *M. bovis*, *M. caprae*, and other closely related mycobacteria. Among them, the main etiological agents of the disease are the highly pathogenic mycobacterial species *M. tuberculosis* and *M. bovis*, though all MTBC pathogens are able to infect humans [4]. Bovine tuberculosis is also a significant zoonotic disease, with transmission to humans occurring primarily through close contact with infected animals or the consumption of contaminated, unpasteurized milk and dairy products.

Among MTBC bacteria, *M. tuberculosis*, *M. bovis* and *M. caprae* are considered to be of the highest importance due to their broad host range and public health significance [5,6]. In cattle, the main cause of the disease (bovine tuberculosis—bTB) is *M. bovis*, but other mycobacteria are also known to infect these animals, such as *M. tuberculosis* and *M. caprae*. Bovine tuberculosis is a chronic granulomatous inflammatory zoonotic disease that has major impacts on cattle health, production, and the livestock industry worldwide due to reduced productivity, high mortality, and carcass condemnation [7]. The disease is considered to be of major importance, affecting the trade of animals and animal products on an international level. According to estimates reported by the WHO, FAO and WOAH, approximately 7% of cattle worldwide may be infected by bovine tuberculosis [5,8]. In addition to cattle and humans, *M. bovis* can infect a wide range of domestic and wild animal species through respiratory or oral transmission. Susceptible hosts include buffaloes, sheep, goats, pigs, horses, camels, cats, dogs, wild ruminants, badgers, deer, and non-human primates [9,10,11]. Co-infections with other respiratory pathogens may occur in cattle populations [12].

Although the majority of developed countries have national programs aimed at controlling the disease by testing herds, pasteurizing milk for human consumption, and conducting meat inspections and animal vaccinations, the disease has not been completely eradicated. According to the consolidated EU Animal Health Law (Regulation (EU) 2016/429), several Member States or zones have achieved official tuberculosis-free status, as defined in the relevant implementing acts. Furthermore, Member States are required to implement official surveillance programs, notify and report disease occurrence, and apply control and eradication measures in accordance with the provisions of Regulation (EU) 2016/429 [13].

Outside the EU, complete eradication has been achieved in Australia [14]. However, in countries where bTB has been eradicated, the maintenance of this status remains challenging, while there is still the possibility of a spillover event of the disease from wildlife sources [15,16]. On the other hand, in low- and medium-income countries, bTB is a major problem for livestock health, welfare, and production, causing considerable economic losses and still representing a serious public health issue [17].

Estimation of the burden of zoonotic bTB is difficult, on account of two main obstacles, i.e., (a) the lack of an organized surveillance program for *M. bovis* as a causative agent of human tuberculosis in bTB-endemic countries associated with low income and high disease burden and (b) the fact that the common laboratory procedures used in the diagnosis of TB are unable to differentiate *M. bovis* from *M. tuberculosis*, attributing all TB cases to *M. tuberculosis* infection. Consequently, the actual burden of zoonotic bTB is probably underestimated [18,19]. The disease is usually transmitted to humans by the consumption of contaminated unpasteurized milk or other dairy products, and less commonly by contaminated meat, while people may also be infected by aerial transmission or through their mucous and skin after handling carcasses of infected animals [5,20,21,22].

In cattle, the most common routes of bTB transmission are either via the respiratory system or through the digestive tract [23,24]. The usual locations of the lesions during respiratory infection are in the nasopharynx, the lungs and the associated lymph nodes. On the other hand, oral transmission is characterized by the ingestion of contaminated feedstuff, water or pastures, with lesions usually appearing in the mesenteric lymph nodes and the intestinal wall [25,26]. In many cases, the disease does not cause any symptoms, highly challenging its control. Symptoms usually appear after a prolonged period of time, the list of the most common symptoms including coughing, dyspnea, appetite and weight loss, weakness, fever, reduction in milk productivity, diarrhea, constipation, infertility and enlargement of lymph nodes. The slow progression of the disease facilitates the spread of the disease by the infected animals to other animals of the herd, before the appearance of any clinical signs [6,27,28].

Diagnosis of bovine tuberculosis in live cattle is mainly based on intradermal tuberculin tests and other immunological assays, including the single intradermal test, comparative intradermal test, interferon-gamma assay, and lymphocyte transformation test [29]. These tests detect a delayed hypersensitivity reaction to mycobacterial antigens in previously sensitized animals [29]. Intradermal tuberculin tests applied for the diagnosis of the disease in cattle are based on purified protein derivatives procured from specific strains of *M. bovis*. In order to avoid cross reactions to customary antigens from non-pathogenic mycobacterial species that may result in false positives, protein derivatives from *M. avium* strains are used in the comparative intradermal tuberculin test [30]. Briefly, for implementation of this test, 72 h after the intradermal injection of tuberculin, skin thickness is measured to determine if any swelling has taken place at the site of the injection [31]. It must be noted that hypersensitivity reactions to tuberculin injection, in general, are developed 3–6 weeks after bTB infection of the animals [32]. Bacteriological culture is regarded as the reference method for the confirmation of bovine tuberculosis, while molecular methods are mostly used as complementary tools for pathogen detection and identification [33].

In Greece, a compulsory bTB eradication program is applied throughout the country, and carried out on all farms at least once a year [34,35]. All cattle over 6 weeks old are routinely examined with the use of a single intradermal tuberculin test, performed by the official state veterinarians. Herds in which no positive animals are detected are classified as disease free. Cattle that react positively to tuberculosis skin test are slaughtered and are subjected to post-mortem examination. The postmortem examination includes visual inspection, palpation and, where necessary, incision of the lungs and lymph nodes.

Carcasses that show tuberculosis lesions are confiscated, and farmers are compensated. Surveillance of the disease also includes both the detection of bTB-like macroscopic lesions during routine inspections conducted in slaughterhouses, and the report of all suspicious lesions for analysis and confirmation, which is made with the performance of bacteriological culture and isolation of bacilli. Treatments on tuberculosis-infected cattle are prohibited. The bTB -positive farms are isolated and no other animals are allowed to enter or leave from the farm. In a farm where the number of infected cattle exceeds 50% of all reared animals, all animals are slaughtered. Hence, any transfer of animals between farms is subject to the prerequisite that the animals intended to be moved must have a negative bTB test one month prior to transfer, and as a general rule, contact between farms with different bTB status is prohibited [34,35].

Although bovine tuberculosis is routinely monitored in Greece, so far there have been no studies describing animal-level positivity and associated risk factors in particular rural areas. The collection and analysis of epidemiological data from infected herds are essential for evidence-based bovine tuberculosis control. Such information can improve understanding of disease distribution, identify factors associated with infection persistence, support targeted surveillance, and assist veterinary authorities in evaluating and refining national control strategies.

The aim of this study was to explore the animal-level bovine tuberculosis positivity patterns among cattle from 30 selected bovine tuberculosis-positive farms in the Regional Unit of Florina, Western Macedonia, Greece, a typical rural area at the borders of Greece with non-EU countries, as well as to explore animal-level and farm-level characteristics associated with positivity within these infected herds. It should be noted that during 2021 and 2022 more than half of the cattle farmed in the Prespes area of Western Macedonia were slaughtered following detection of bovine tuberculosis under the national eradication program, resulting in substantial socioeconomic consequences at both the regional and national levels. To the best of our knowledge, this is the first systematic investigation of bovine tuberculosis positivity patterns in cattle farms in Greece.

## 2. Materials and Methods

### 2.1. Study Area

**Figure 1:**
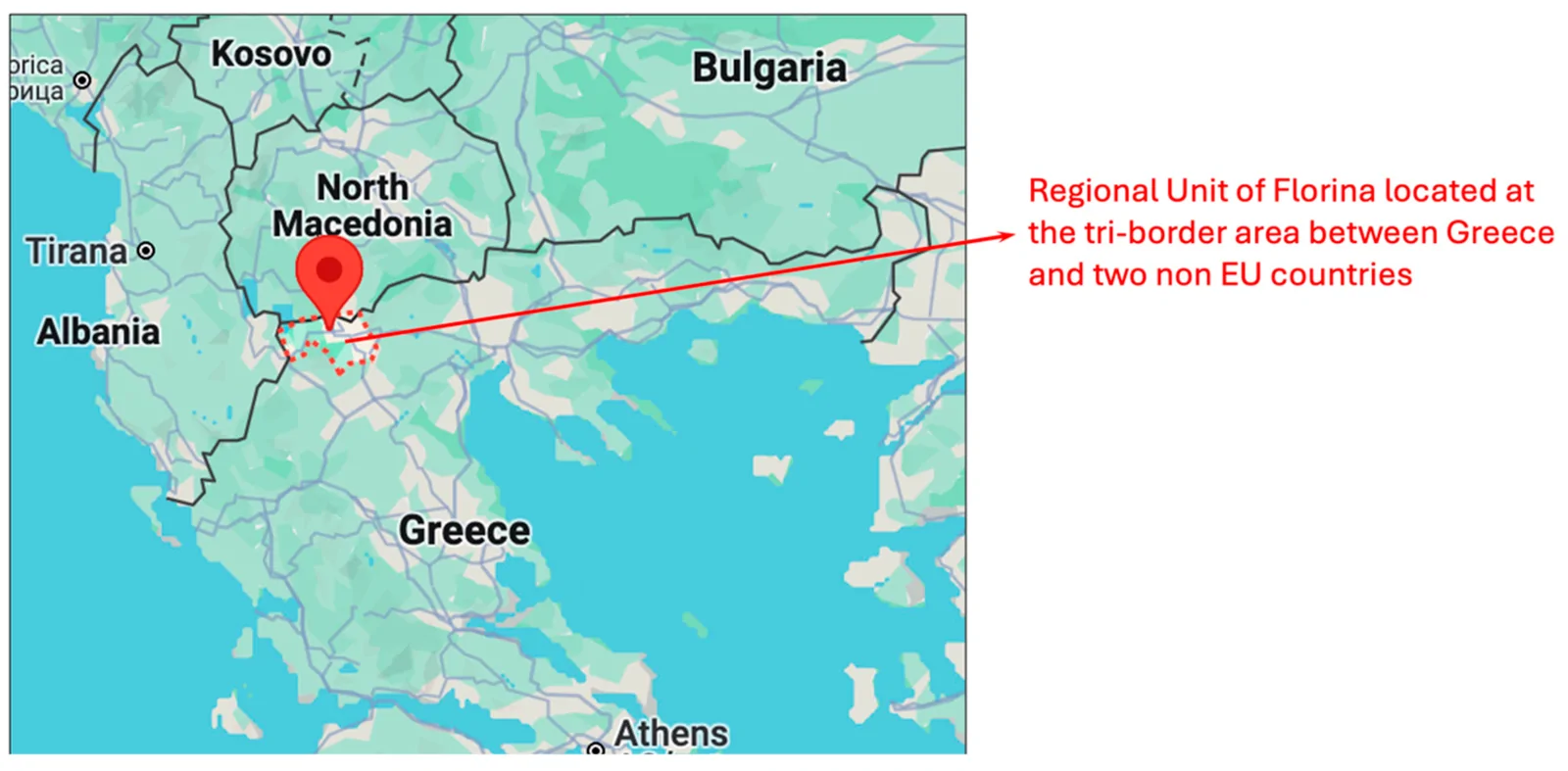
The Regional Unit of Florina, from which the tuberculosis-related data were collected, located at the gateway of the EU to the Western (non-EU) Balkan countries. The Figure was made by the authors, using Google Maps JavaScript API and Microsoft PowerPoint v. 16 software.

The study was conducted in the Regional Unit of Florina, located in the Region of Western Macedonia, Greece. It represents the least densely populated regions in mainland Greece, accounting for less than 3% of the country’s population. Livestock farming plays a crucial role in the economy and regional development of the Regional Unit of Florina, whereas the mountainous topography supports grazing in pastures that are characterized by low productivity but high plant biodiversity and species richness [36]. It is worth noting also that the Region of Western Macedonia is the entrance gateway of Greece and the European Union to the Western Balkans (Figure 1).

The cattle population of the Regional Unit of Florina typically ranges between 10,000 and 12,000 animals, with normal changes owing to births, deaths, slaughter, and animal movements. Florina was particularly selected on account of numerous recent bovine tuberculosis cases observed, representing a rural livestock-producing area where extensive and semi-extensive cattle farming systems are still common. The farmed breeds are mainly Limousin animals, Friesian-Holstein, and to a much lesser extent, Brown Swiss Cattle crossbred cattle.

### 2.2. In Vivo Animal Testing for Tuberculosis

Cattle from 30 selected farms originating from the Regional Unit of Florina were investigated to estimate apparent animal-level bTB positivity and investigate associated risk factors. Animal-level positivity was the main outcome, whereas farm-level positivity was also recorded.

Both farms and the individual cattle (set as the experimental units) were selected using convenience sampling based on the national surveillance program. The minimum required total sample size was calculated by the formula *n* = (1.96^2^ × *P* × *Q*)/*L*^2^, where 1.96 represents the *z*-score for a 95% level of confidence, *P* = 0.50 is the expected prevalence of positive animals, *Q* = 0.50 is the expected prevalence of negative animals (1 − *P*), and *L* = 0.05 is the allowable error. Given the absence of similar previous studies in the area, a conservative expected prevalence (*P* = 0.50) was used, resulting in a minimum required sample size of 385 animals, which was substantially exceeded in our sampling scheme. Particularly, a total of 1464 cattle were examined, originating from 30 dairy, meat production or mixed farms, using a single intradermal test.

The 30 farms included in the study were determined through the official registry of cattle holdings maintained by the Veterinary Directorate of Western Macedonia. Farms were approached based on geographical accessibility and farmer willingness to participate. It should be clarified that the results reflect the sampled farms and should not be considered representative of all cattle farms in the Region of Western Macedonia.

The study was conducted during the routine annual statutory bovine tuberculosis surveillance program in the Regional Unit of Florina, Western Macedonia. Farm recruitment was based on participation in routine surveillance activities rather than on a specific epidemiological investigation. Although bovine tuberculosis had previously been detected in the study area, the present study was not initiated as part of a formally declared outbreak investigation. All eligible cattle older than six months within each participating farm were tested. Of the 47 farms approached, 30 agreed to participate (63.8% enrolment) and, finally, data collection and analyses took place between March and July 2023.

The investigated farms were determined through the official regional bovine tuberculosis surveillance program, whereas all eligible animals present at each visit in the farms were selected from the registry of active cattle. Skin testing followed the national eradication protocol, using the single intradermal cervical test, which is considered more sensitive than the caudal fold test. Bovine PPD (CZV Bovine Tuberculin PPD, CZ Veterinaria S.A., Spain; batch numbers 2205584 [2023] and 2201183 [2022]) was administered intradermally at the mid-cervical region (border of the anterior and middle third of the neck) at a volume of 0.1 mL, after clipping the site to facilitate accurate reading. Skin fold thickness was measured immediately before injection and repeated at 72 h, following the national practice. Interpretation thresholds were ≤2 mm (negative), 2–4 mm (inconclusive), and >4 mm or clinical signs (positive), while no severe interpretation was applied. Testing was performed by trained official veterinarians without blinding. Inconclusive reactors were retested after ≥42 days and classified as positive if failed to produce a negative result. Positive reactors, as well as animals reclassified as positive after retesting, were slaughtered following official veterinary decision and movement permit issuance. In cases where epidemiological assessment suggested potential false positive reactions, a comparative intradermal test (bovine and avian PPD) could be applied according to national procedures, although this was not required for the animals included in the present dataset. All animals used in the study were over the age of six months, and were reared under an intensive, semi-intensive or extensive system.

Animals exhibiting clinical signs compatible with tuberculosis and those ones receiving treatment for acute disease, as well as recently vaccinated animals, were excluded to minimize potential interference with the skin test response. All positive animals in the intradermal tuberculin test were slaughtered according to the national eradication program regulations, and underwent post-mortem examination. The examination included visual inspection, palpation and, where necessary, incision of the lungs and associated lymph nodes. Lymph nodes presenting gross lesions compatible with bovine tuberculosis were sampled and proceeded to molecular analysis. One lymph node sample was collected from each animal and submitted for PCR confirmation.

### 2.3. Molecular Confirmation of Positive Samples and Investigation of the Presence of Other Bacteria

A PCR was applied to detect *Mycobacterium* spp., as, apart from skin test positivity, such presence was not confirmed before sample collection. The VetMAX *M. tuberculosis* Complex PCR Kit was utilized for postmortem molecular confirmation of *Mycobacterium* spp. presence, in an Eco 48 PCR max (Cole-Parmer Antylia Scientific, Vernon Hills, IL, USA). Approximately 50 mg of lymph node tissue sample were obtained using sterilized scalpel and forceps and subjected to DNA isolation utilizing the NucleoSpin Tissue kit (Macherey-Nagel, Duren, Germany) according to the manufacturer’s instructions. The quality and concentration of the isolated DNA were measured in a Q5000 UV-Vis spectrophotometer (Quawell Technology, Sunnyvale, CA, USA) and DNA was accordingly diluted at 40 ng/μL. Five μL of isolated DNA were mixed with Mix MTBC, whereas a Positive control, an Extraction control (mock-purified sample) and a No-template control were included in each reaction. The real-time PCR regime consisted of two initial steps of 50 °C for 2 min and 95 °C for 5 min, followed by 40 cycles of 95 °C for 10 s and 60 °C for 30 s, with fluorescence set at the last step.

Since co-infection may be common, we also performed a second molecular technique to examine the presence of other pathogens too. In an attempt to explore the secondary infection of tissues by other bacteria in all lymph node samples collected, a conventional PCR was performed in the positive samples, using the primer pair 27f–1492r [37], which amplifies a conserved region of the 16r RNA gene in a great variety of bacteria but has been shown to fail amplification in members of the genus *Mycobacterium* [38]. PCRs were carried out in 20 μL total reaction volumes containing 10 μL FastGene Taq 2x Ready Mix (NIPPON Genetics, Dueren, Germany), 0.6 pmol of each primer, 1.5 μL isolated DNA and ultrapure water up to the final volume. PCR conditions consisted of an initial denaturation step at 94 °C, followed by 38 cycles at 94 °C for 20 s, 50 °C for 30 s, and 72 °C for 1 min, ending with a final extension step at 72 °C for 5 min. Amplified products were purified using the NucleoSpin Gel and PCR Clean-up Mini kit (Macherey-Nagel, Dueren, Germany) and bidirectionally sequenced in an ABI-Prism 3730 automatic sequencer. The software MEGA 11 was used for molecular genetic analysis of the sequences in comparison to the most closely related haplotypes obtained from the GenBank database, based on searches in the BLAST (http://www.ncbi.nlm.nih.gov/BLAST) tool on the NCBI website.

### 2.4. Determination of Risk Factors

Data concerning farm management and type, as well as animal-related characteristics, were collected using a questionnaire given to each farmer. The questionnaire was administered by the official veterinarian responsible for bovine tuberculosis surveillance in the study area, through face-to-face interviews with farmers. Responses were recorded during farm visits and subsequently evaluated by an author of the study who served as a public veterinarian. Data collection was performed within the framework of the official bovine tuberculosis surveillance program; therefore, neither the interviewer nor the farmers were blinded to herd status. Information regarding animal origin (born on the farm or purchased) was not systematically collected and was therefore unavailable for analysis.

The results of the questionnaire were evaluated by an author of the study who is a public veterinarian surveilling the farms. The variables recorded were sex (male or female); age group (≤1, 2–3, 4–6, or >6 years old); husbandry type (intensive or semi-extensive); farm system type (dairy, beef, or mixed); herd size (small: 1–10, medium: 11–50, large: more than 50, as defined by Tulu et al. [26]; breed (Friesian-Holstein or Limousin); biosecurity and hygiene status (low or high); contact with other herds (yes or no); contact with wildlife (yes or no); history of TB (yes or no); keeping at night (indoors or outdoors); feeding (straw, alfalfa, intensive grazing or exclusive grazing); grazing type (communal or non-communal); proximity to border area [short distance (less than or equal to 5 km from the border area) or long distance (more than 5 km from the border area)]; introduction of new animals within last two years, as proposed by Islam et al. [10], and other animals reared on the farm (sheep, goats, pigs, and poultry) [10]. Information regarding *Mycoplasma bovis* infection was not obtained through the questionnaire but was determined separately through laboratory analysis, as described below.

### 2.5. Statistical Analysis

The TB status (negative or positive) was the dependent variable, while the animal-level and herd-level characteristics were the independent variables. Descriptive statistics were calculated for the total sample and based on the TB status. Particularly, for the animal-level and herd-level characteristics, frequencies and apparent positivity, with the 95% confidence interval (CI) of Wilson, were summarized by category.

To evaluate associations between the characteristics and the TB status, a multilevel logistic regression (Generalized Linear Mixed Model–GLMM) was applied. Considering the hierarchical structure of the data, the herd (farm 1–30) was defined as random effect (to account for within-farm correlation), while risk factors were defined as fixed effects. The probability distribution was Binomial, and the link function was Logit. Also, the analysis was performed in R software (version 4.4.1) using the “lme4” package and the “glmer” function, with the “bobyqa” optimizer for model convergence.

The level of significance was set at 5%. The statistical analyses were performed using the statistical software program IBM SPSS Statistics v26.0 and the programming language R v4.3.2. The R code used for the GLMM models, the data dictionary (Table S2), and several Supplementary Tables and Figures of data analysis are presented in the Supplementary Material Section.

## 3. Results

All 30 sampled farms contained at least one positive animal, a finding that reflects only the apparent herd-level positivity among the sampled farms (100%) and does not necessarily represent herd-level positivity. Because the sampling was not population-based, no regional prevalence or morbidity estimates were calculated.

**Figure 2:**
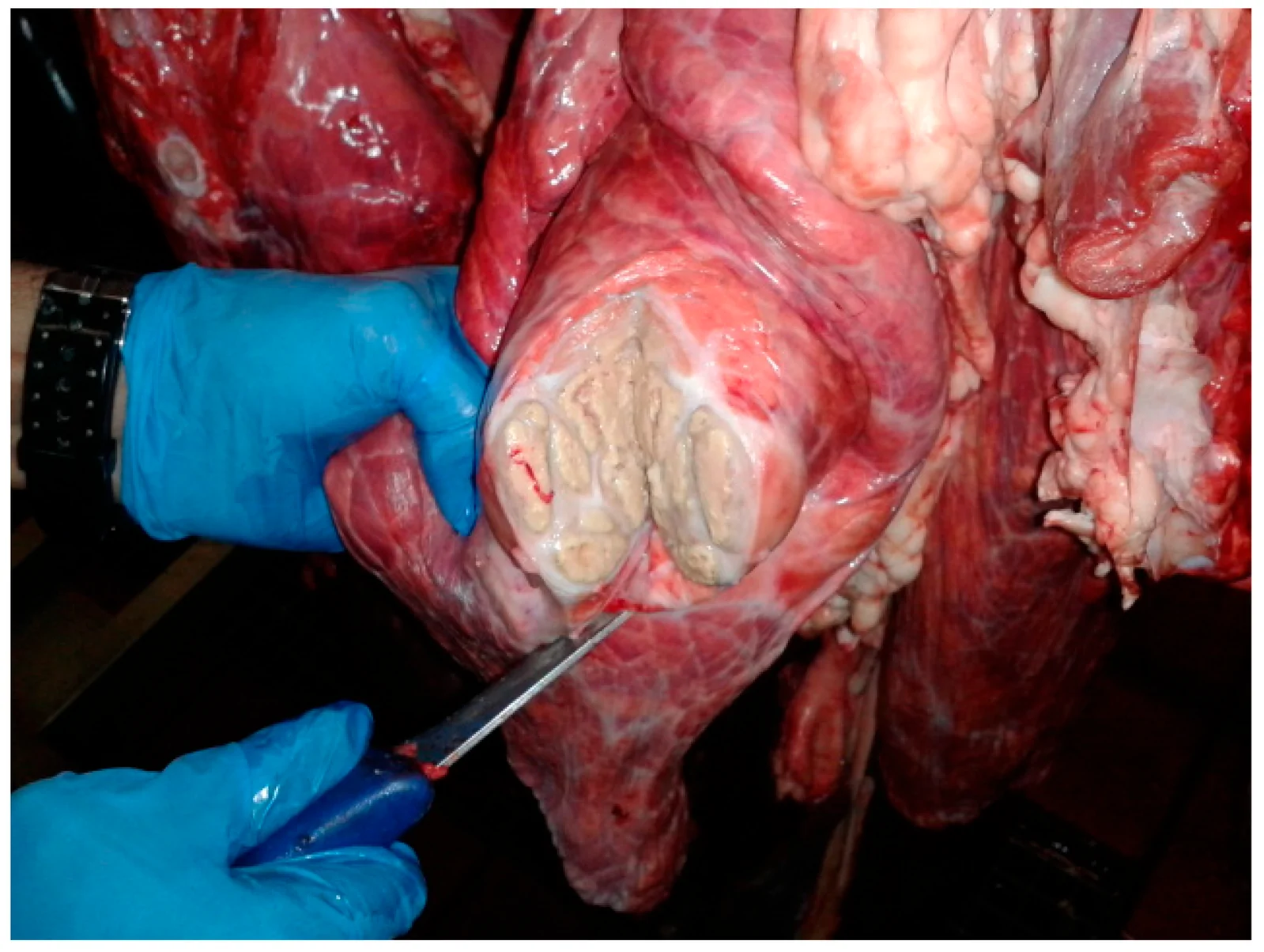
Cattle lungs with tuberculosis lesions. The circumscribed, yellowish, granulomatous nodule 2–20 mm in diameter is clearly shown. At first, it appears as a barely visible, gray, transparent nodule which, although it sometimes heals, usually expands, most often slowly, and its center becomes necrotic (cheesy necrosis). The Figure photograph was taken by the authors.

The total sample size was 1464 cattle. Of these, 901 (61.5%) were scored as TB-negative, while 563 (38.5%) were scored as TB-positive, resulting in an apparent animal-level positivity of 38.5% within the sampled farms (Table 1). All MTBC-positive cattle were subsequently subjected to postmortem examination, whereas no skin test-negative animals were examined postmortem, in accordance with the national bovine tuberculosis eradication program. Gross TB-like lesions were observed in all examined animals (Figure 2). Histopathology, Ziehl–Neelsen staining, and mycobacterial culture were not performed, as the national eradication program mandates PCR confirmation only. Following application of the MTBC real-time PCR assay, positive fluorescence signals were detected in all DNA samples obtained from tissues exhibiting gross (macroscopic) lesions compatible with bovine tuberculosis, confirming the MBTC infection in all animals with gross lesions. Therefore, complete agreement was observed between postmortem detection of TB-like lesions and MTBC PCR positivity in the animals examined. This finding is explained by the study design, in which only skin test-positive cattle underwent postmortem examination and PCR testing. At the herd level, 11 farms were characterized as heavily infected, with more than 50% of animals testing positive and with all positive animals from these farms subsequently slaughtered (Table S1).

**Figure 3:**
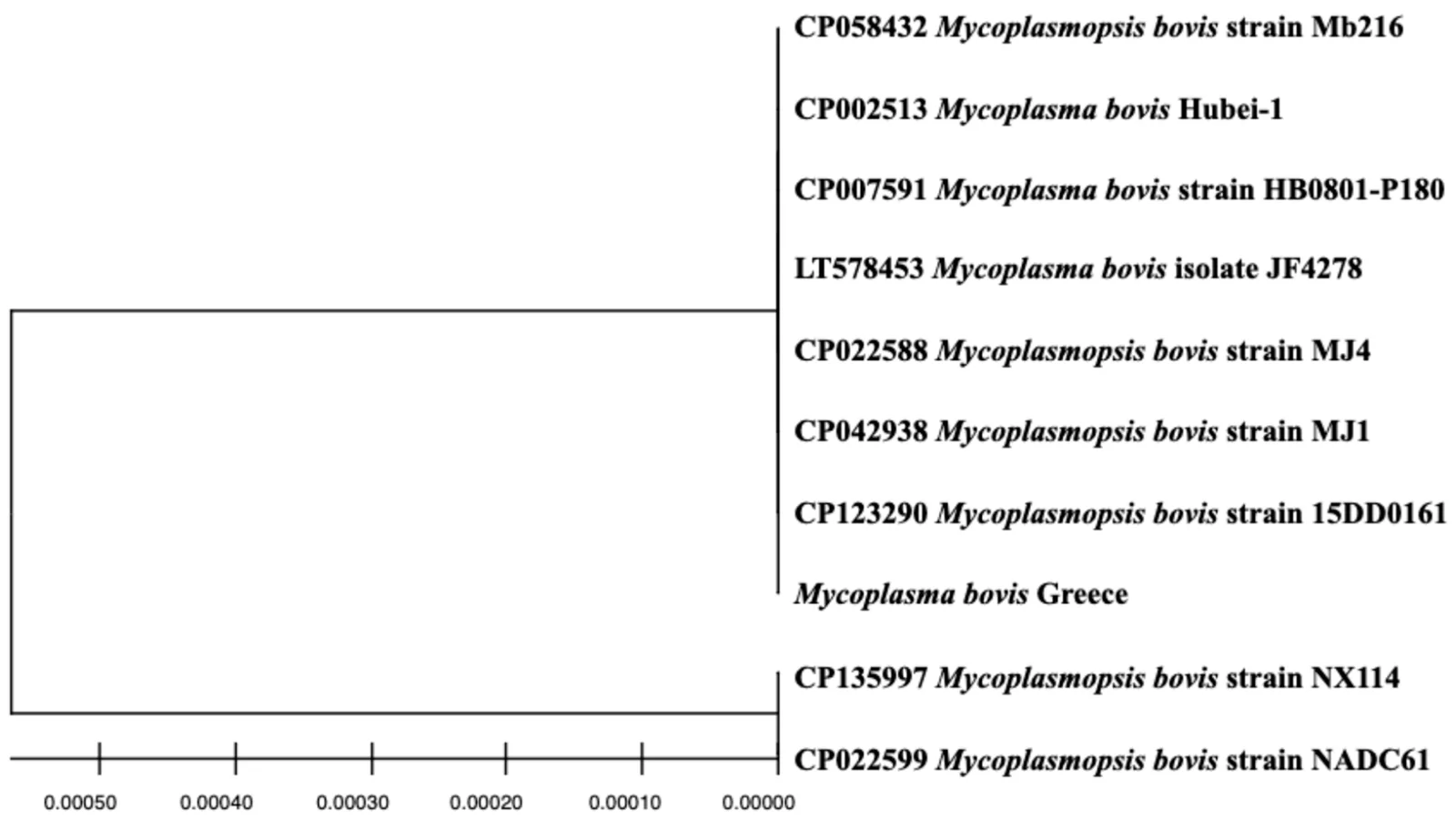
Identification of *Mycoplasma bovis* based on identical genetic relationships with conspecific sequences obtained from the GenBank database.

The PCR targeting broad-range bacterial species amplified sequences identical with *Mycoplasma bovis* in 12 of 563 MTBC-positive cattle (2.13%) (Figure 3), which thus were attributed to this species. Because MTBC-negative cattle were not tested, no clear association between *Mycoplasma bovis* detection and tuberculosis status could be evaluated.

Regarding the animal-level characteristics (Table 1), 174 male and 389 female cattle were recorded as positive, with 45% and 36% apparent positivity. Higher apparent positivity was presented in younger cattle (65% to 96%), than in older ones (9%). Regarding breed, 186 positive cattle were Friesian-Holstein, with 22% apparent positivity, while 377 were Limousin, with 63% positivity.

Regarding the herd-level characteristics (Table 2), 155 cattle of intensive type and 408 cattle of semi-extensive type were recorded as positive, resulting in 29% and 44% animal-level positivity, respectively. In addition, higher animal-level apparent positivity was observed in beef farm systems (58%), than in dairy (24%) and mixed ones (29%). Regarding contact with other herds and/or wildlife, higher animal-level positivity was presented in cattle which were in contact with wildlife (≥44%). Concerning TB history, higher animal-level apparent positivity was shown in cattle which had a history of TB (41%). Furthermore, cattle which were kept outdoors at night had higher prevalence (58%) than those which were kept indoors at night (28%). Regarding the presence of other animals, higher animal-level apparent positivity was found in farms with dogs, poultry and sheep or only dogs (43%), compared to farms with dogs, poultry and cats (38%) or only dogs and cats (7%). Finally, investigating the proximity to border area, three farms (farms 14, 17, and 19) and, by extension, 24 positive cattle, were far from borders (with 16% animal-level apparent positivity), while all the other farms and, by extension, 539 positive cattle, were close to borders (with 41% animal-level apparent positivity).

In addition, all cattle were associated with communal grazing and were characterized by low biosecurity and hygiene status. Due to the lack of variability in these factors, they were excluded from further statistical analysis.

To obtain more information about the effects of various characteristics on TB status, GLMM was conducted. Sex and age were retained as animal-level characteristics potentially associated with animal susceptibility or exposure. Husbandry type was retained as a broad farm-management indicator, whereas highly correlated farm-management variables describing overlapping aspects of the production system were not simultaneously included. Proximity to the border was retained because geographical location was a predefined study objective. Also, all characteristics were assessed for multicollinearity using the Generalized Variance Inflation factor (GVIF), adjusted for degrees of freedom (df). Characteristics representing highly overlapping aspects of farm management were not simultaneously included in the final model. Husbandry type was retained as a broader indicator of the management system, while the selection of fixed effects also considered the level of measurement, biological relevance, and the objectives of the study. Particularly, the characteristics of farm system type, breed, keeping at night, TB history, presence of other animals, feeding, and contact with other herds and/or wildlife were excluded from the final model.

The co-infection with *M. bovis* could not be included in the model, due to the nature of the data, where co-infection was observed exclusively in TB-positive cattle (zero variance in the negative group), leading to a state of complete separation that precluded model convergence.

Therefore, the fixed effects of the model were sex, age, husbandry type, and proximity to borders. Herd (farm 1–30) was included as a random effect to account for the hierarchical structure of the data and the clustering of animals within farms, while the dependent variable was TB status (negative vs. positive). The model was fitted by maximum likelihood (Laplace Approximation) using the “bobyqa” optimizer. The results of the corresponding GLMM are presented in Table 3.

ORs and 95% CIs were obtained from the maximum-likelihood GLMM. The GLMM results showed that the sex was not a statistically significant factor associated with apparent animal-level positivity [*β* = 0.35, OR = 1.42, 95% CI (0.84, 2.39), *p* = 0.189]. Regarding age, the older age groups (2–3, 4–6, and >6 years) showed significantly lower odds of apparent animal-level TB positivity compared to the youngest age group (≤1 year) [*β* ≤ −3.26, ORs ≤ 0.04, *p* < 0.001]. Husbandry type was not associated with statistically significant differences in the odds of apparent animal-level TB positivity between semi-extensive and intensive systems [*β* = 0.35, OR = 1.42, 95% CI (0.08, 25.70), *p* = 0.813]. Finally, while a close distance from border areas presented a higher odds ratio compared to far distances to borders, this finding was not statistically significant [*β* = 1.37, OR = 3.92, 95% CI (0.05, 331.92), *p* = 0.546]. However, the estimate was highly imprecise because only three farms were classified as being far from the border. The estimated variance of the herd-level random intercept was 12.24 (SD = 3.50).

**Figure 4:**
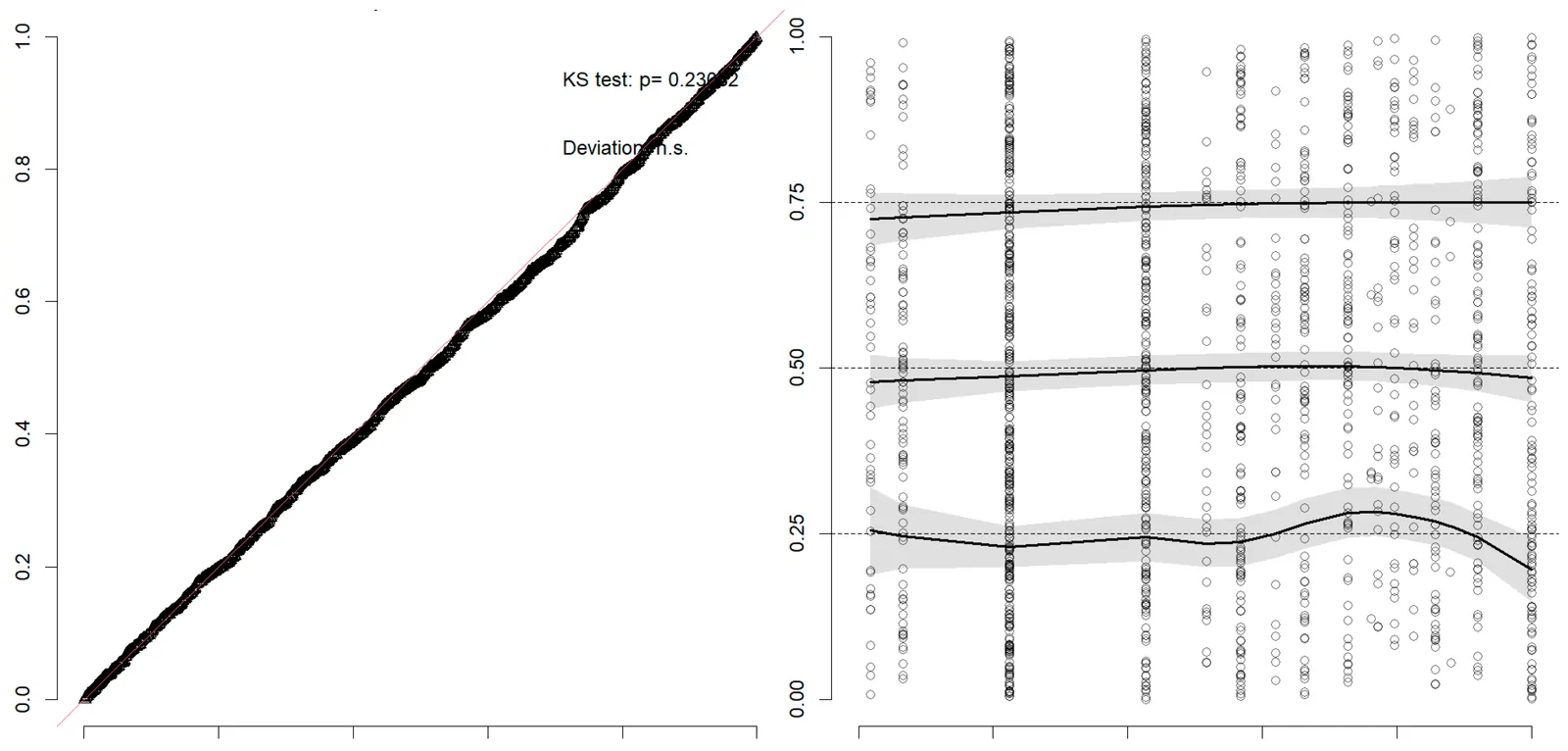
Diagnostic plots of the GLMM using simulated quantile residuals (DHARMa). The Q–Q plot (**left**) and the residuals vs. predicted plot (**right**) demonstrate a good model fit.

The Intraclass Correlation Coefficient (ICC) was calculated as σherd2σherd2+π23, where *π* = 3.14 and $\sigma_{herd}^{2}$ is the random effect (herd) variance. In this case, ICC was 0.79, indicating substantial within-herd correlation and considerable between-herd heterogeneity in the probability of TB positivity. Thus, accounting for herd-level clustering was important because animals within the same herd could not be treated as statistically independent observations. Also, DHARMa residual diagnostics did not detect significant deviations in the assessed residual patterns (Figure 4).

Overall, after accounting for clustering at the herd level, age was the only characteristic showing evidence of an association with apparent animal-level TB positivity. The substantial between-herd variability indicates that the observed positivity patterns were strongly influenced by herd-level clustering. Therefore, crude differences in apparent positivity across animal-level and herd-level characteristics should be interpreted descriptively rather than as independent risk-factor associations.

The extremely low adjusted ORs and wide 95% CIs observed for the older age categories were consistent with quasi-separation arising from the near-complete positivity observed in the youngest reference group (≤1 year) (Figure S1). Particularly, sex, husbandry type, and proximity to the border remained statistically non-significant, whereas the older age categories remained strongly associated with lower odds of apparent TB positivity compared with the youngest age group. Thus, the main conclusions of the primary analysis were unchanged in the sensitivity analysis. These estimates should therefore be interpreted with caution. Furthermore, the initial GLMM model converged without warnings, and no singular fit was detected.

## 4. Discussion

Bovine tuberculosis is a severe re-emerging disease affecting livestock, wildlife, and humans. On a global scale, the disease is responsible for substantial economic losses in animal production, poses a threat to the ecosystem and accounts for about 3.1% of all human TB cases [39,40]. In many parts of the world, cattle are the main hosts of *M. bovis* in nature, but due to the multi-host characteristics of the disease, eradication of bTB is extremely complicated [11]. In a few developed countries, such as in Australia and Japan, the disease has been eliminated, but in the majority of the developed and developing countries of the world bTB is still endemic [41]. Transmission is influenced by various factors including environmental factors, bacterial and host characteristics and measures implemented for the control of the disease [42].

To the best of our knowledge, this is the first study in Greece to systematically investigate MBTC-positivity and associated risk factors in cattle. Additionally, *Mycoplasma bovis* was detected in a few MBTC-positive animals, for the first time in co-infection with MBTC. Concerning the epidemiological significance of this finding, it should be noted that it remains unclear, with no strict conclusions being drawn regarding a potential association between *Mycoplasma bovis* and bovine tuberculosis.

Despite the limited geographic focus that reduces the broader generalizability of our findings, we trust that the discussed inferences offer considerable merit as the first systematic investigation of apparent animal-level positivity and its associated risk factors among cattle in Greek farms conducted in the country as well as in the Balkan region. Interestingly, although it is not statistically significant, there is a noteworthy trend of geographic location, with mostly affected farms being in close proximity to the border areas adjacent to non-EU countries, which occasionally follow different regulations.

It should be highlighted that we included herds identified as positive through routine surveillance in a region with persistent bovine tuberculosis occurrence, which is highly important for extensive cattle farming. Historical herd-level infection records were not consistently available and therefore previous infection status could not be evaluated, representing a limitation of the present investigation. Although the sampling design does not allow estimation of regional prevalence, differences in apparent positivity according to animal- and farm-level characteristics were observed, providing descriptive evidence of epidemiological patterns within the investigated herds.

Specifically, our study revealed that 38.5% (563/1464) of the examined animals were positive. Previous studies have demonstrated that the sensitivity and specificity of the single intradermal tuberculin test, postmortem lesion detection, and PCR are not perfect. Therefore, complete agreement among these diagnostic approaches would not generally be expected. In the present study, however, all SIT-positive animals exhibited lesions compatible with tuberculosis and were PCR-positive for MBTC. This high level of agreement may be attributable to the targeted examination of reactor animals from already-infected herds and the selection of tissues with characteristic gross lesions. The observation that all SIT-positive animals included in the study presented visible lesions is noteworthy, as lesion detection is generally reported to have imperfect sensitivity. The high concordance observed may reflect the advanced stage of infection among reactor animals selected for slaughter and postmortem examination. PCR confirmation in all lesion-positive animals represents a high level of diagnostic agreement. This may be also attributable to targeted sampling of lymph nodes presenting characteristic tuberculosis lesions rather than random population-level sampling.

In previous research studies conducted in other regions and countries, individual animal prevalences ranged from 4.3 to 39.3%, where herd prevalences ranged from 16.7 to 58.5% [9,19,26,40,43]. Similar findings have also been reported in several European countries where bovine tuberculosis remains endemic in specific regions despite long-standing control programs, including Spain, Ireland and the United Kingdom [13]. Likewise, a study conducted in central China reported animal prevalence of 18.65%, and herd-level prevalence of 93.48% [41], whereas, in Bangladesh, Islam et al. (2020) reported animal and herd prevalences of 11.3% and 45.6% respectively, and in Malawi, researchers revealed that 9.95% of slaughtered cattle had pathological lesions suggestive of bTB [10]. Differences in prevalence rates among research studies could be attributed to, among others, different geographical locations, socioeconomic conditions, study designs, diagnostic tools applied, farm management and production systems, herd sizes and genotype [19].

The univariate analysis identified several animal- and management-related risk factors associated with TB positivity, including breed, production type, husbandry system, proximity to the border and co-infection with *M. bovis*. However, after adjustment for the hierarchical structure of the data, the farm-level random effect accounted for a substantial proportion of the observed variability (ICC = 0.79), indicating that these factors operate within a strong herd-specific context. Therefore, the following discussion refers primarily to the crude epidemiological associations observed in the study, while taking into account the dominant influence of herd-level clustering.

Based on our findings, MBTC infection is highly influenced by geographical area, co-infection with *Mycoplasma bovis*, breed, farm type (beef production) and husbandry type (Table 1). However, the last two factors are mostly attributed to the particular type of farming applied in the area. According to this type, cattle are reared freely, grazing for six to seven months per year, whereas for the rest of the year, they are kept inside the farm. During this 6–7-month period, they may encounter cattle from other farms, even from neighboring countries, in line with the statistically significant correlation of the geographic location with proximity to the cross-border area (Table 1).

Although descriptive analyses showed differences in apparent positivity between geographical areas, proximity to the national border was not significantly associated with apparent positivity after accounting for herd-level clustering. Therefore, our findings do not support border proximity as an independent risk factor for bovine tuberculosis test positivity. Nevertheless, farms located near the border may still need further investigation because factors such as animal movements, grazing practices, and wildlife interactions could influence disease transmission. No data on animal movements, molecular typing, or cross-border epidemiological links were available in this study and therefore more secure inferences cannot be drawn apart from the trend for farms located closer to the cross-border area, which could also be attributed to other demographic features [44,45]. The findings of the present study should be interpreted within the broader European framework for bovine tuberculosis control, including the classification of countries and regions according to their official tuberculosis-free status. Although a substantial number of European Union Member States have achieved Officially Tuberculosis Free status, bovine tuberculosis remains a challenge in certain regions, highlighting the heterogeneous distribution of the disease across Europe [13].

The EU countries employ various monitoring and control measures, depending on specific risk thresholds, while non-EU countries with less stringent protocols could potentially act as disease reservoirs. European Regulation (EU 2016/429) specifies the prevention, detection and response measures for the control of infectious diseases, with a particular emphasis on cross-border coordination.

Until recently, transmission of bTB was believed to be facilitated mainly via direct contact among hosts. In fact, most of *M. bovis* shedding takes place via aerosols and, to a limited extent, through feces, urine, saliva or milk. Hence, close contact is the main route of transmission between infected and susceptible animals [46]. Nevertheless, more recently, the hypothesis that contamination of the environment might play a role in the preservation of the disease has been suggested in several studies proposing potential transmission of bacilli via indirect contact [46,47]. The survival period of bacilli in the environment ranges from a few days up to two years, depending on factors such as temperature, exposure to sunlight and humidity. Generally, survival of bacilli is facilitated by a dark, cool, moist and not directly exposed to sunlight environment, and in usual temperatures, they can survive in slurry and soil for at least a 6-month period [2]. In pastures where cattle are grazing, there is a high probability of environmental contamination through animal feces, droplets or nasal mucus. This manure that remains untreated could probably increase the risk of indirect transmission of the disease [48]. Contamination through the shedding of *M. bovis* bacilli from infected animals has already been described, apart from cattle, in wild animals, as in badgers, deer and boars [47,48]. A characteristic of the positive farms was the fact that these animals were grazing in a nearby field where they were in close contact with animals of neighboring herds. This fact could probably enhance the possibility of disease dissemination, as has been indicated in previous published studies. More specifically, Campbell et al. (2020) stated that grazing and contact with neighboring herds is considered a risk factor for the transmission of various infectious diseases among cattle, like the infectious bovine rhinotracheitis, foot and mouth disease, bovine viral diarrhea and bTB [49]. Similarly, communal grazing and close contact with neighboring herds has been recognized as a factor that favors high prevalence rates of bTB infection [50]. In addition, the use of pastures is highlighted as a factor that increased the risk of bTB infection in a study conducted in Spanish cattle herds [51].

The assumption that age is positively associated with bTB infection has been proposed in several studies, such as that by Kapalamula et al. (2023), when it was reported that older animals appear to have a higher risk of infection, as a result of prolonged exposure to bacilli, reactivation of latent infections, and physiological decrease in immunity status [52]. A positive association of bTB prevalence and age was also recorded in a study conducted in cattle in Ethiopia [40].

In our study, infection was correlated to young animals (aged less than one year). This finding differs from most published studies and may be related to the specific epidemiological conditions of the investigated herds. Youngstock can be infected at any age, mainly via the respiratory or the digestive route, and as the course of disease is relatively slow, these animals can spread bTB bacilli to other animals in the herd, or to other animals that are in close contact, before the manifestation of clinical disease [53]. Immune response of calves to *M. bovis* could be impacted by various stress factors, such as nutritional and transport-related stress. Their immune system, though competent, is still naïve and it is possible that young animals are more susceptible and develop more severe disease compared to older animals [32]. Therefore, early exposure to infected animals and the increased susceptibility of young calves may explain the higher prevalence observed in animals less than one year old in the present study. Additionally, although transfer of animals was not recorded, as it is often not reported officially, the young age in our case could be related with buying and selling of young animals among farms that are more easily infected.

Susceptibility to bTB has also been previously associated with genetic variation, such as in Irish cattle, suggesting that Holstein dairy cows were more resistant to disease and Simmental and Charolais beef breeds were reported more susceptible to the disease [54]. Susceptibility to bTB was investigated also by Vordermeier et al. (2014) in a study in Ethiopia, where it was supported that “exotic” Friesian-Holstein breeds appeared to be more susceptible than indigenous African zebu breeds, which also presented less pathology [55]. Association of breed with disease susceptibility was reported by Wangmo et al. (2024) as well, who found more positive cases in crossbred cattle that in indigenous breeds, a fact that could be related to the genetic composition of these animals [56]. Similarly, a positive association of bTB cases with breed, and more specifically with crossbred dairy cattle, was inferred in Ethiopia [57]. Selection of breeds towards disease resistance has been therefore proposed as an additional measure for the control and eradication of bTB [58].

Herd size has also been recognized as a risk factor for bTB infection in previous published studies and a positive association among herd size and disease prevalence was identified. Ghebremariam et al. (2018) stated that in the cases in which farmers increased herd size, there was a higher probability of *M. bovis* cattle-to-cattle transmission, probably due to overcrowding [59]. A similar correlation was proposed by Sichewo et al. (2020), where bTB-positive herds were associated with herd sizes greater than 15 cattle [60]. In Uruguay, farm size was associated positively with *M. bovis* transmission. In this study, researchers stated that large-herd transmission is facilitated by the fact that there is probably a high density of animals at the paddock and prolonged periods of waiting in the pre-milking area. Moreover, due to limited sensitivity of the diagnostic intradermal tests, there is a risk of introducing infected animals to the farm, an event that is more possible to occur in large than small herds, as the number of shipments was positively associated with the size of the farm. Interestingly, most of the bTB-positive farms in that study were located in a national region with a higher density of dairy farms and close to the national borders [61].

In addition, *Mycoplasma bovis* co-infection was detected in a limited number of farms with *Mycobacterium* infection. Co-infections with other microorganisms are common in cases of bTB in cattle. *Pasteurella multocida*, *Mannheimia haemolytica*, bovine respiratory syncytial virus, bovine herpes virus 1, bovine viral diarrhea virus and parainfluenza virus type 3 are among the most often encountered [62]. To the best of our knowledge, here we report for the first time *Mycoplasma* spp. co-infection in cattle. Co-infections have been pointed out as potential modulators of immune responses of infected animals to various bacterial infections, such as bTB. Specifically, for bTB, it has been suggested that several pathogens, including the *Bovine Viral Diarrhea Virus* (BVDV), *Fasciola hepatica* and the *Mycobacterium avium* subsp. *Paratuberculosis* (MAP), can implicate disease epidemiology. This could be associated with an exacerbation of clinical disease, potentially leading to increased host susceptibility and to increased bacterial shedding that could lead to increased *M. bovis* transmission [63,64]. However, since co-infection was detected in only 12 of the 563 MBTC-positive animals examined (2.13%), interpretation of this finding should be made with caution. Therefore, the prevalence of *Mycoplasma bovis* in the overall cattle population could not be determined. Moreover, *Mycoplasma bovis* is a well-recognized respiratory pathogen of cattle and may also be detected in apparently healthy animals [65,66,67,68]. Therefore, its detection in the present study does not automatically indicate a causal role in bovine tuberculosis-associated lesions. *Mycoplasma bovis* infections in cattle have been associated with several clinical manifestations, such as pneumonia, mastitis, arthritis and keratoconjunctivitis [65]. These infections have been considered responsible for suppression of hosts’ immune responses, producing synergistic infections with other microorganisms and facilitating the development of various chronic conditions, mainly due to increased susceptibility to other pathogens [66,67,68]. An example of such synergism has been demonstrated in pneumonia cases in beef calves, where co-infection of *Mycoplasma bovis* and *Pasteurellaceae* family pathogens was reported [69].

Although positive animals have been found without a statistically significant correlation with management conditions (hygiene status) of the farm, this was due to the absence of a comparator high biosecurity status. Poor management conditions could generally increase the possibility of animal infection. Farms that have been managed poorly have a higher probability of being positive for tuberculosis than those with good hygiene/management conditions. Association of poor management conditions with bTB positivity has also been reported in previous studies. Tulu et al. (2021) reported a significant association between hygiene status and bTB positivity at the herd level, stating that in farms with poor management conditions and hygiene status, a favorable environment may be created for bacilli proliferation and transmission [26]. Similarly, in a study conducted in cattle in Ethiopia, herd positivity was strongly related to management conditions, documenting that improvement of hygiene conditions could subsequently improve the bTB status of the farms [70]. An analogous association was reported by Romha et al. (2018), who stated that poor sanitation status could favor the prolonged persistence of bacilli in the farms [4]. A strong association of bTB positivity and herd management practices has also been reported by Kemal et al. (2019), where farms with poor management practices presented a 3.66 times higher probability of being positive to the disease, compared to farms that implemented good management practices [19].

Finally, in extensive and semi-extensive systems, interactions between cattle and wildlife may be of high epidemiological importance, depending on local ecological conditions. Wildlife species have been documented as potential reservoirs of *M. bovis* in several regions worldwide, and their presence can contribute to complex epidemiological patterns at the livestock–wildlife interface. Although wildlife surveillance data were not available for the present study area, acknowledging this interface is important for interpreting apparent positivity in rural grazing systems as well as for designing future monitoring approaches [71].

## 5. Conclusions

This study documented high MBTC positivity in cattle farms in Florina, Greece, indicating substantial variations in positivity between farms. Age-related differences in positivity were observed, with younger and older animals showing different positivity levels; however, these findings should be interpreted with caution given the cross-sectional nature of the study. Overall, the study highlights the need for more representative surveillance and molecular epidemiological investigations to improve understanding of MBTC-infection patterns in the region and to support evidence-based control strategies. Future studies should include representative sampling and further investigation of farm-level and wildlife-related factors that may influence infection dynamics in similar production systems.

## Figures and Tables

**Table 1:** Frequencies and the apparent positivity (%), with 95% confidence intervals (CI), of the categories for each animal-level characteristic (Sex, Age and Breed), relative to the total sample size and by TB status (negative vs. positive).

Animal-Level Characteristics	Total (*n* = 1464)	TB-Negative Animals (*n* = 901)	TB-Positive Animals (*n* = 563)	Apparent Positivity (%)	95% CI
**Sex**					
Male	388	214	174	44.8%	(40.0–49.8)
Female	1076	687	389	36.2%	(33.3–39.1)
**Age**					
≤1	226	9	217	96.0%	(92.6–97.9)
2–3	164	40	124	75.6%	(68.5–81.5)
4–6	221	78	143	64.7%	(58.2–70.7)
>6	853	774	79	9.3%	(7.5–11.4)
**Breed**					
Friesian–Holstein	863	677	186	21.6%	(18.9–24.4)
Limousin	601	224	377	62.8%	(58.8–66.5)

**Table 2:** Descriptive distributions of animal-level apparent positivity (%), with 95% confidence intervals (CI), according to herd-level characteristics (husbandry type, farm system type, contact with other herds/wildlife, TB history, keeping at night, feeding, and proximity to border area).

Herd-Level Characteristics	Total (*n* = 1464)	TB-Negative Animals (*n* = 901)	TB-Positive Animals (*n* = 563)	95% CI
**Husbandry type**				
Intensive (no grazing in common pasture) (Farms; *N* = 9)	541	386	155	(25.0–32.6)
Semi-extensive (grazing in common pasture) (Farms; *N* = 21)	923	515	408	(41.0–47.4)
**Farm system type**				
Dairy (Farms; *N* = 14)	377	287	90	(19.8–28.4)
Beef (Farms; *N* = 7)	546	228	318	(54.1–62.3)
Mixed (combination) (Farms; *N* = 9)	541	386	155	(25.0–32.6)
**Contact with other herds**				
Yes (Farms; *N* = 20)	906	511	395	(40.4–46.8)
No (Farms; *N* = 10)	558	390	168	(26.4–34.0)
**Contact with wildlife**				
Yes (Farms; *N* = 6)	529	224	305	(53.4–61.8)
No (Farms; *N* = 24)	935	677	258	(24.8–30.5)
**TB history**				
Yes (Farms; *N* = 1)	29	17	12	(25.5–59.3)
No (Farms; *N* = 29)	1435	884	551	(35.9–40.9)
**Presence of other animals**				
Dogs (Farms; *N* = 17)	1093	618	475	(40.5–46.4)
Dogs and cats (Farms; *N* = 2)	174	162	12	(4.0–11.6)
Dogs, poultry and cats (Farms; *N* = 10)	190	117	73	(31.8–45.5)
Dogs, poultry and sheep (Farms; *N* = 1)	7	4	3	(15.8–75.0)
**Keeping at night**				
Indoors (Farms; *N* = 24)	935	677	258	(24.8–30.5)
Outdoors (Farms; *N* = 6)	529	224	305	(53.4–61.8)
**Feeding**				
Straw, alfalfa, intensive grazing (Farms; *N* = 24)	935	677	258	(24.8–30.5)
Exclusive grazing (Farms; *N* = 6)	529	224	305	(53.4–61.8)
**Proximity to border area**				
Close (less than 5 km) (Farms; *N* = 27)	1310	771	539	(38.5–43.8)
Far (more than 5 km) (Farms; *N* = 3)	154	130	24	(10.7–22.1)

**Table 3:** Results of multilevel logistic regression (GLMM) [apparent positivity (positive/total), estimate (*β*), standard error (SE), adjusted odds ratio (OR) with 95% confidence interval (CI), and *p*-values]. The reference categories (ref) are also presented, as well as the variance and standard deviation (SD) of the random effect.

Characteristic	Apparent Positivity	Estimate (*β*)	SE	Adjusted OR	95% CI	*p*-Value
**Sex**						
Male (ref)	174/388					
Female	389/1076	0.35	0.27	1.42	(0.84–2.39)	0.189
**Age**						
≤1 (ref)	217/226					
2–3	124/164	−3.26	0.45	0.04	(0.02–0.09)	<0.001
4–6	143/221	−5.41	0.59	0.004	(0.001–0.014)	<0.001
>6	79/853	−14.20	1.38	6.8 × 10^−7^	(4.5 × 10^−8^–1.0 × 10^−5^)	<0.001
**Husbandry type**						
Intensive (ref)	155/541					
Semi-extensive	408/923	0.35	1.48	1.42	(0.08–25.70)	0.813
**Proximity to border area**						
Far (ref)	24/154					
Close	539/1310	1.37	2.26	3.92	(0.05–331.92)	0.546
**Random Effect**	**Variance**	**SD**				
**Herd (Farm 1–30)**	12.24	3.50				

## Data Availability

Data are included within the article

## Supplementary Materials

The following supporting information can be downloaded at: https://www.mdpi.com/article/10.3390/ani16182947/s1, Table S1: Frequencies and apparent positivity of each herd (farm 1–30), relative to the total sample size and by TB status (negative vs. positive); Table S2: Data dictionary (level and coding); The complete R code; Figure S1: Apparent animal-level TB positivity according to age group. Bars represent the proportion of cattle classified as TB-positive within each age group (0 = Negative TB status and 1 = Positive TB status). The marked gradient across age groups is presented descriptively and does not account for clustering of animals within herds or other covariates.
