# Supplementary material for: Bovine Tuberculosis in a Rural Greek Area: Within-Herd Prevalence and Risk Factors in 30 Tuberculosis Skin Test-Positive Herds
Source: Animals (Basel). 2026 Sep 19;16(18):2947. doi: 10.3390/ani16182947 (PMC13603457; doi:10.3390/ani16182947)
Supplement: Supplementary file 1 [file animals-16-02947-s001.zip › animals-4456664-supplementary.pdf]

Supplementary material

In Table S1, the frequencies and apparent positivity of each farm were presented, regarding the total sample size and by TB status.

Table S1. Frequencies and apparent positivity of each herd (farm 1-30), relative to the total sample size and by TB status (negative vs positive).

**Herd**	**Total** (n=1,464)	**TB negative animals** (n=901)	**TB positive animals** (n=563)	**Apparent Positivity**
Farm 1	17	4	13	0.76
Farm 2	6	4	2	0.33
Farm 3	98	74	24	0.24
Farm 4	55	30	25	0.45
Farm 5	97	72	25	0.26
Farm 6	66	48	18	0.27
Farm 7	103	78	25	0.24
Farm 8	77	52	25	0.32
Farm 9	15	12	3	0.20
Farm 10	18	12	6	0.33
Farm 11	12	8	4	0.33
Farm 12	7	4	3	0.43
Farm 13	87	83	4	0.05
Farm 14	87	79	8	0.09
Farm 15	36	17	19	0.53
Farm 16	16	6	10	0.63
Farm 17	37	24	13	0.35
Farm 18	26	25	1	0.04
Farm 19	30	27	3	0.10
Farm 20	4	3	1	0.25
Farm 21	18	12	6	0.33
Farm 22	1	-	1	1.00
Farm 23	12	3	9	0.75
Farm 24	10	-	10	1.00
Farm 25	100	35	65	0.65
Farm 26	12	-	12	1.00
Farm 27	98	50	48	0.49
Farm 28	142	70	72	0.51
Farm 29	109	49	60	0.55
Farm 30	68	20	48	0.71

In Table S2, the data dictionary was presented.

Table S2. Data dictionary (level and coding).

Variable	Level	Coding
TB status	Animal	0 = Negative, 1 = Positive
Herd	Herd	1 = Farm 1 – 30 = Farm 30
Sex	Animal	1 = Male, 2 = Female
Age	Animal	1 = Unknown, 2 = ≤1 y.o., 3 = 2-3 y.o., 4 = 4-6 y.o., 5 = >6 y.o.
Breed	Animal	1 = Holstein, 2 = Limousin
Husbandry type	Herd	1 = Mixed, 2 = Intensive, 3 = Semi-extensive
Farm type	Herd	1 = Mixed, 2 = Dairy, 3 = Beef
Biosecurity/Hygiene status	Herd	1 = Poor/Low
Contact with other herds	Herd	1 = Yes, 2 = No, 3 = No (trade of animals,vehicles and feed)
Contact with wildlife	Herd	1 = No, 2 = Yes
TB history	Herd	1 = No, 2 = Yes
Presence of other animals	Herd	1 = Dogs, 2 = Dogs and cats, 3 = Dogs, poultry and cats, 4 = Dogs, poultry and sheep
Keeping at night	Herd	1 = Indoors, 2 = Outdoors
Feeding	Herd	1 = Straw, alfalfa, and intensive grazing, 2 = Exclusive grazing
Grazing type	Herd	1 = Communal
Proximity to borders	Herd	1 = Short distance (≤5 km), 2 = Long distance (> 5 km)
M. bovis co-infection	Animal	0 = Negative, 1 = Positive

The complete R code was presented below.

```
# Install and load packages
install.packages(c("haven", "car", "binom", "lme4", "Matrix", "blme", "performance", "DHARMa", "dplyr",
"ggplot2"))
library (haven)
library (car)
library(lme4)
library (Matrix)
library(blme)
library(performance)
library(DHARMa)
library(binom)
library(dplyr)
library(ggplot2)

# Import data
data=read_sav("C:/Users/…./TB_dataset.sav")

# Convert variables to factors
data$TB_status = as.factor(data$TB_status)

vars_to_factor = c("Sex", "Age", "Breed", "Husbandry_type", "Farm_type", "Biosecurity_Hygiene_Status",
        "Contact_with_other_herds", "Contact_with_wildlife", "TB_history", "Presence_of_other_animals",
```

```
                "Keeping_at_night", "Feeding", "Grazing_type", "Proximity_to_borders", "Co_infection")
data[vars_to_factor] = lapply(data[vars_to_factor], as.factor)
data$Herd = as.factor(data$Herd)

# Apparent positivity with Wilson 95% CI
wilson_table = function(data, variable) {

  x = data[[variable]]

  levels_x = levels(x)

  results = lapply(levels_x, function(level) {

    idx = !is.na(x) & x == level

    total = sum(idx)
    positive = sum(data$TB_status[idx] == 1)

    ci = binom.confint(
      x = positive,
      n = total,
      method = "wilson"
    )

    data.frame(
      Variable = variable,
      Category = level,
      Total = total,
      Positive = positive,
      Apparent_positivity = positive / total * 100,
      CI_lower = ci$lower * 100,
      CI_upper = ci$upper * 100
    )
  })

  do.call(rbind, results)
}
table1_CI = rbind(
  wilson_table(data, "Sex"),
  wilson_table(data, "Age"),
  wilson_table(data, "Breed")
)
table1_CI

table2_CI = rbind(
  wilson_table(data, "Husbandry_type"),
  wilson_table(data, "Farm_type"),
  wilson_table(data, "Contact_with_other_herds"),
  wilson_table(data, "Contact_with_wildlife"),
  wilson_table(data, "TB_history"),
  wilson_table(data, "Presence_of_other_animals"),
  wilson_table(data, "Keeping_at_night"),
  wilson_table(data, "Feeding"),
```

```
  wilson_table(data, "Proximity_to_borders")
)
table2_CI

# Multicollinearity
vif_check = glm(TB_status ~ Sex + Age + Breed + Husbandry_type + Farm_type + Contact_with_other_herds +
             Contact_with_wildlife + TB_history + Presence_of_other_animals + Keeping_at_night + Feeding + _
              Proximity_to_borders,
               data = data, family = binomial)
vif_results <- car::vif(vif_check)
vif_results

if (is.matrix(vif_results)) {

  gvif_results = data.frame(
    Predictor = rownames(vif_results),
    GVIF = vif_results[, "GVIF"],
    df = vif_results[, "Df"],
    Adjusted_GVIF =
      vif_results[, "GVIF"] ^
      (1 / (2 * vif_results[, "Df"]))
  )

  print(gvif_results)

} else {

  gvif_results = data.frame(
    Predictor = names(vif_results),
    VIF = as.numeric(vif_results)
  )

  print(gvif_results)
}

# Initial GLMM
model = glmer(TB_status ~ Sex + Age + Husbandry_type + Proximity_to_borders + (1 | Herd),
                 data = data,
                 family = binomial(link = "logit"),
                 control = glmerControl(optimizer = "bobyqa", optCtrl = list(maxfun = 2e5)))
summary(model)

# Initial adjusted ORs and Wald 95% CIs
exp(fixef(model))
exp(confint(model, method="Wald"))

# Penalized/Bayesian GLMM
model_pen= glmer(TB_status ~ Sex + Age + Husbandry_type + Proximity_to_borders + (1 | Herd),
                 data = data,
                 family = binomial(link = "logit"),
                 control = glmerControl(optimizer = "bobyqa", optCtrl = list(maxfun = 2e5)))
summary(model_pen)
```

```
# Penalized fixed-effects estimates
beta_pen = fixef(model_pen)

# Penalized adjusted ORs and 95% CIs
OR_CI_pen <- data.frame(
  Predictor = names(beta_pen),
  OR = exp(beta_pen),
  CI_lower = CI_pen_OR[, "Lower"],
  CI_upper = CI_pen_OR[, "Upper"]
)
OR_CI_pen

# DHARMa residuals (initial model)
simulationOutput = simulateResiduals(fittedModel = model, plot = TRUE)
simulationOutput = simulateResiduals(fittedModel = model)
plot(simulationOutput)
dev.off()
par(mar=c(1,1,1,1))
plot(simulationOutput)

# ICC
performance::icc(model)
performance::icc(model_pen)

# Plot of apparent animal-level TB positivity according to age
ggplot(data, aes(x = factor(Age), fill = factor(TB_status))) +
  geom_bar(position = "fill") +
  facet_wrap(~ Herd, ncol = 6) +
  labs(x = "Age group", y = "Proportion", fill = "TB status") +
  theme_bw()
```

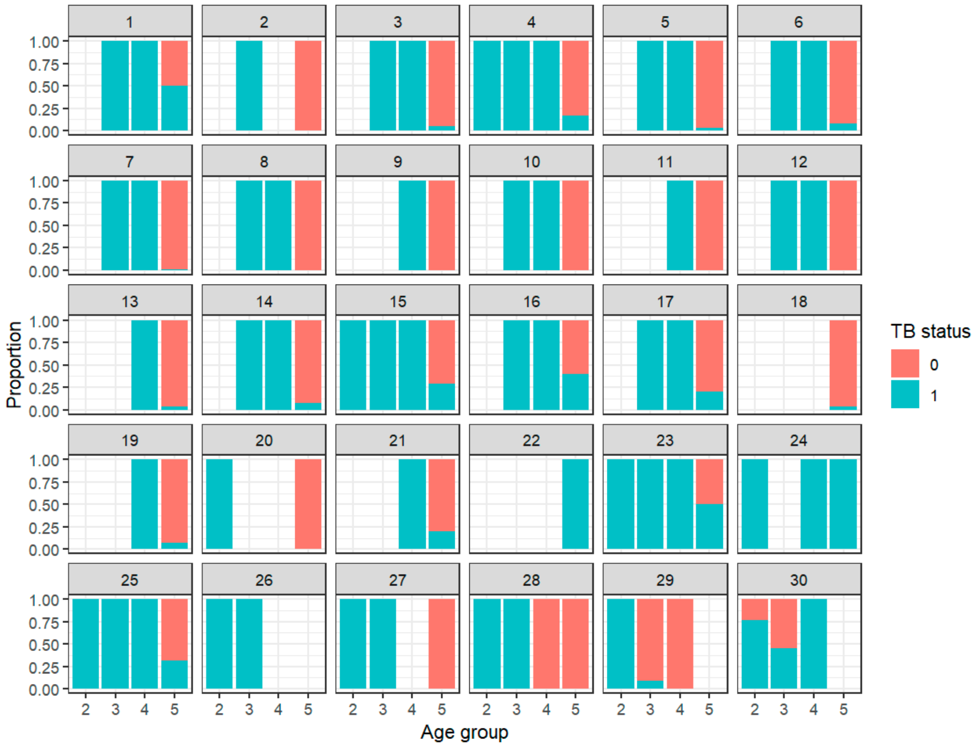

Figure S1. Apparent animal-level TB positivity according to age group. Bars represent the proportion of cattle classified as TB-positive within each age group (0 = Negative TB status and 1 = Positive TB status). The marked gradient across age groups is presented descriptively and does not account for clustering of animals within herds or other covariates.
